# Suicidal Ideation and Attempts in Trichotillomania

**DOI:** 10.1016/j.psychres.2023.115245

**Published:** 2023-05-06

**Authors:** Jon E. Grant, Madison Collins, Eve Chesivoir, Samuel R. Chamberlain

**Affiliations:** a Department of Psychiatry & Behavioral Neuroscience, University of Chicago, Pritzker School of Medicine, Chicago, IL, USA; b Department of Psychiatry, Faculty of Medicine, University of Southampton, UK; and Southern Health NHS Foundation Trust, Southampton, UK

**Keywords:** trichotillomania, suicidality, comorbidity

## Abstract

Trichotillomania is characterized by chronic pulling out of one’s hair. Given the negative sequelae of trichotillomania, we examined rates of suicidal ideation and suicide attempts. Of the 219 adults (mean age = 29.5 years; 88% female) recruited, 40 (18.3%) reported lifetime suicidal ideation, and 5 (2.3%) reported a lifetime suicide attempt. Those with histories of suicidal ideation were significantly more likely to have major depressive disorder. Our findings suggest that suicidal ideation and attempts are common in trichotillomania and support the idea that comorbid depression should be considered a risk factor for suicidality.

## 1 Introduction

Trichotillomania is characterized by the failure to resist impulses to pull out one’s hair often resulting in noticeable hair loss (American Psychiatric Association, APA, 2013). Trichotillomania is classified as an obsessive-compulsive and related disorder in the Diagnostic and Statistical Manual Version 5 (DSM-5) (APA, 2013). The condition is frequently associated with poor self-esteem, depression, psychosocial dysfunction, and overall lower quality of life (Woods et al., 2006). More specifically, approximately 45% of people with trichotillomania struggle with comorbid major depressive disorder (Grant et al., 2020). Given the negative consequences associated with trichotillomania, and the elevated rates of co-occurring major depressive disorder, we would expect that rates of suicidal ideation may also be elevated in this population, and yet we have found no data on rates of suicidal ideation or suicide attempts among people with trichotillomania.

To address this unmet need, the present study assessed suicidal ideation and suicide attempts in a large sample of adults with trichotillomania using a secondary analysis of pooled research studies. We hypothesized that both suicidal ideation and suicide attempts would be elevated in individuals with trichotillomania compared to normative data from the US general population.

## 2 Methods

### 2.1 Participants

Adults with trichotillomania who had participated in a range of research studies were included. Inclusion criteria for all studies were: current DSM-5 trichotillomania, written informed consent, and the ability to understand the study and the consent form. Exclusion criteria were: bipolar I disorder, schizophrenia, or an alcohol/substance use disorder in the preceding three months. Data from baseline visits were used for the current study. The sample was recruited from metropolitan areas in the USA.

After receiving a complete description of the study, participants provided written informed consent. All procedures involving human subjects were approved by the Institutional Review Board at the University of Chicago. The authors assert that all procedures contributing to this work comply with the ethical standards of the relevant national and institutional committees on human experimentation and with the Helsinki Declaration of 1975, as revised in 2008.

### 2.2 Assessments

Participants were assessed for age, biological sex at birth, self-reported gender, and racial-ethnic identity. Suicidality was assessed using the Columbia Suicide Severity Rating Scale (C-SSRS) (Posner et al., 2011), a valid and reliable semi-structured clinical interview consisting of subscales assessing current and lifetime suicidal ideation and suicidal behavior.

Other assessments included: Massachusetts General Hospital Hairpulling Scale (MGH-HPS), a 7-item self-report scale (total scores range from 0 to 28) assessing hair pulling over the past seven days (Keuthen et al., 1995); Clinical Global Impression-Severity scale (CGI) (Guy, 1976) to assess overall reported symptoms as well as observations of hair loss; Mini International Neuropsychiatric Interview (MINI; Sheehan et al., 1998) or the Structured Clinical Interview for DSM-IV (First et al., 1995) to examine co-occurring psychiatric disorder; Quality of Life Inventory (QOLI) (Frisch et al., 2005), a self-report scale assessing satisfaction in sixteen domains of life; and the Barratt Impulsiveness Scale 11 (BIS-11) (Patton et al., 1995).

### 2.3 Statistics

We present descriptive characteristics of the sample including percentage of those with lifetime suicidal ideation and lifetime suicide attempts. Furthermore, the sample was divided into two subgroups according to the lifetime presence or absence of suicidal ideation: trichotillomania with lifetime suicidal ideation (score ≥ 1 on the suicidal severity subscale) compared to trichotillomania without any lifetime suicidal ideation (score = 0). To analyze the sociodemographic and clinical features associated with lifetime suicidal ideation, groups were compared using independent sample t-tests for continuous variables and Pearson's chi-square tests for categorical variables. Statistical significance was defined as *P* < .05, Bonferroni corrected for the number of measures at the level of type of measurement.

## 3 Results

The study comprised 219 adults with trichotillomania (mean age = 29.5 [SD=7.63) years [range 18 to 64 yrs]; 88.0%) female). Of 219 participants, number and percentages of people in different racial-ethnic categories were: Caucasian 174 (79.5%), Black 23 (10.5%), Asian 13 (5.9%), Latino/Hispanic 5 (2.3%),), and Other/Mixed Race 4 (1.8%).

Of the 219 participants, 40 (18.3%) reported lifetime suicidal ideation, and 5 (2.3%) reported a lifetime suicide attempt. When we compared those with lifetime suicidal ideation to those without lifetime suicidal ideation, we found no significant differences with respect to age (*t*(211) = 0.699, *p* = 0.49), gender (X^2^(2) = 0.60, *p* = 0.74), or race/ethnicity (X^2^(6) = 12.12, *p* = 0.06).

In terms of comorbidity, those with lifetime suicidal ideation were significantly more likely to have co-occurring major depressive disorder (62.5% compared to 34.7%; χ^2^(1) = 10.48; p<.001) (see Table 1). The groups did not significantly differ with respect to other psychiatric comorbidities (all p>0.10).

The participants with lifetime suicidal ideation did not significantly differ from those without suicidal ideation with respect to trichotillomania symptom severity (MGH-HPS scores of 18.3 [SD = 3.52] compared to 18.4 [SD = 3.53], respectively; *t*(209) = .14, p=.891), overall global mental health severity (CGI scores of 4.4 [SD = 0.59] compared to 4.4 [SD = 0.69] respectively; *t*(209) = .49, p=.627), quality of life (QOLI t-scores of 31.5 [SD = 25.1] compared to 42.91 [SD = 14.0] respectively; *t*(81) = 1.8 p=.075), or impulsivity (BIS total scores of 62.7 [SD = 12.3] compared to 61.4 [SD = 12.1] respectively; *t*(181) = -.64, p=.526).

## 4 Discussion

This study is the first that we are aware of that examined suicidal ideation and attempts in adults with trichotillomania. We found that the rate of lifetime suicidal ideation was almost one in five (18.3%) and that approximately 2% had lived through one or more suicide attempt(s) in their lifetimes. To put these data into a larger context, a study exploring suicidal behaviors across 17 countries using data from the World Health Organization World Mental Health Survey Initiative, found a cross-national lifetime prevalence of 9.2% for suicidal ideation and 2.7% for suicide attempts (Nock et al., 2008). In a National Comorbidity Survey (1990-1992) in the USA, using a nationally representative sample, 13.5% of individuals reported lifetime suicidal ideation and 4.6% reported a lifetime attempt (Kessler et al., 1999).

These data for trichotillomania are interesting when also compared to data regarding suicidal ideation and attempts in other obsessive-compulsive spectrum disorders. A meta-analysis of suicidal ideation in obsessive-compulsive disorder (OCD) found that at least one person out of ten with OCD experiences one or more suicide attempt(s) during their lifetime (pooled prevalence rate 13%), while nearly half of individuals with OCD have experienced suicidal ideation (pooled prevalence rate of lifetime suicidal ideation 47%) (Pelligrini et al 2020). An earlier meta-analysis found that comorbid disorders, increased severity of obsessions, feelings of hopelessness and past history of suicide attempts were associated with worsening levels of suicidality in OCD (Angelakis et al., 2015). Another study of suicide attempts in adults with OCD found that 14.6% of the sample (n=425) reported at least one suicide attempt during their lifetime (Dell’Osso et al., 2018). Finally, a recent meta-analysis of OCD and related disorders found that for body dysmorphic disorder, the pooled prevalence of lifetime suicide attempts and lifetime suicidal ideation were, respectively, 35.2% and 66.1%, whereas for grooming disorders (these studies only included skin picking), the pooled prevalence of lifetime suicide attempts and current suicidal ideation were 13.3% and 40.4%, respectively (no data was available for lifetime suicidal ideation) (Pelligrini et al., 2021).

Based on our data, trichotillomania appears to have a lower rate of both lifetime suicidal ideation and suicide attempts compared to certain other obsessive-compulsive spectrum disorders. One possible explanation for these differences could be that the symptoms of trichotillomania, while impairing, are less likely to result in suicidal ideation or attempts as compared to the symptoms of OCD or BDD. Given the reported low quality of life and depression, however, it will be important to determine what protective factors against suicidality people with trichotillomania possess that others in the OCD spectrum may not have.

Suicidal ideation in trichotillomania was significantly associated with having comorbid major depressive disorder. Having said that, it is also important to note that approximately 40% of those with suicidal ideation did not have comorbid major depressive disorder. It is likely that those with greater general psychopathology who have both depression and trichotillomania are also more likely to have suicidal ideation. It is not possible to unentangle the complex relationship between suicidality, depression and hair pulling based on our limited data. Some of these participants might have been depressed and suicidal due to their pulling, others may have been depressed and suicidal independent of their pulling, and others may have had suicidal ideation independent of pulling and of depressive symptoms. Another way of interpreting these results, however, is not that depression is a risk factor but instead suicide is an independent problem and thus a shared outcome of a subset of trichotillomania patients and a subset of some depressed patients. Regardless, these data do suggest that everyone with trichotillomania should be screened for major depressive disorder and suicidal ideation. There are important limitations to this study. First, the sample for the present study was recruited from a diverse group of research studies and the recruitment method may have varied slightly. Second, the findings of this study may not be generalizable to people with trichotillomania in the general community, or to specific clinical practice situations, since all participants were part of various research studies. Third, we only used the Columbia Suicide Rating Scale to examine suicidality, and did not include a measure of disability, and therefore future research may consider adding other measures of interest. Fourth, we did not ask what if any relationship hair pulling had to suicidal ideation. Finally, the study enrolled 80% Caucasians and therefore minority racial-ethnic groups were underrepresented, relative to the general population, which may affect the generalizability of the results. Prior work indicates that suicidality in general maybe higher in particular minority racial-ethnic groups, notably in relation to indigenous populations (Troya et al., 2022), highlighting the need to study this issue in future trichotillomania research.

## Figures and Tables

**Table 1:** Psychiatric Co-Morbidities by Lifetime Suicidal Ideation ^a^

	Lifetime Suicidal Ideation (N = 40)	No Lifetime Suicidal Ideation (N = 173)
MDD	25 (62.5%) ***	60 (34.7%) ***
OCD	1 (2.5%)	11 (6.4%)
ADHD	2 (5%)	12 (6.9%)
Any Anxiety Disorder	7 (17.5%)	45 (26.0%)
Any Eating Disorder	2 (5%)	3 (1.7%)
Any SUD	0 (0%)	9 (5.2%)

*Note*. A Bonferroni correction was applied to account for multiple comparisons, leaving the critical p-value at 0.007 (0.05/7).

a Data are presented in N (%) form unless otherwise indicated.

*** *p* < .001
